# CDC27 Promotes Tumor Progression and Affects PD-L1 Expression in T-Cell Lymphoblastic Lymphoma

**DOI:** 10.3389/fonc.2020.00488

**Published:** 2020-04-23

**Authors:** Yue Song, Wei Song, Zhaoming Li, Wenting Song, Yibo Wen, Jiwei Li, Qingxin Xia, Mingzhi Zhang

**Affiliations:** ^1^Department of Oncology, The First Affiliated Hospital of Zhengzhou University, Zhengzhou, China; ^2^The Academy of Medical Science of Zhengzhou University, Zhengzhou, China; ^3^Lymphoma Diagnosis and Treatment Center of Henan Province, Zhengzhou, China; ^4^Department of Pathology, The Affiliated Cancer Hospital of Zhengzhou University, Henan Cancer Hospital, Zhengzhou, China

**Keywords:** CDC27, T-cell lymphoblastic lymphoma, PD-L1, cell cycle, APC/C

## Abstract

T-lymphoblastic lymphoma (T-LBL) is a rare hematological malignancy with highly aggressive, unique clinical manifestations, and poor prognosis. Cell division cycle 27 (CDC27) was previously reported to be a significant subunit of the anaphase-promoting complex/cyclosome. However, the specific functions and relevant mechanisms of CDC27 in T-LBL remain unknown. Through immunohistochemistry staining, we identified that CDC27 was overexpressed in T-LBL tissues and related to tumor progression and poor survival. Functional experiments demonstrated that CDC27 promoted proliferation *in vivo* and *in vitro*. Further experiment suggested the role of CDC27 in facilitating G1/S transition and promoting the expression of Cyclin D1 and CDK4. Then the effect of CDC27 in inhibiting apoptosis was also identified. Furthermore, we found a positive correlation between the expression of CDC27 and Programmed death ligand-1 (PD-L1) by immunohistochemistry staining. The interaction between CDC27 and PD-L1 was also proved by western blot, luciferase gene reporter assay and immunofluorescence. Taken together, our results showed that CDC27 contributes to T-LBL progression and there is a positive correlation between PD-L1 and CDC27, which offers novel perspectives for future studies on targeting CDC27 in T-LBL.

## Introduction

Lymphoblastic lymphoma is a rare type of non-Hodgkin lymphoma (NHL) with biological characteristics similar to acute lymphoblastic leukemia (ALL). On the basis of the origin of tumor cells, it is primarily classified into B-cell lymphoblastic lymphoma and T-cell lymphoblastic lymphoma (T-LBL) (1). Because of the progressive improvements in therapeutic strategies, especially the wide application of targeted therapies, the prognosis of B-cell lymphoma has improved obviously. However, the clinical progression of T-LBL is rapid and the prognosis is poor. The standard NHL-like treatment plan is less effective with higher probability of recurrence and metastasis. Because of the rarity of the disease and limited materials for research, there is also a lack of knowledge about the molecular genetics and effective targeted therapies for T-LBL (2). Therefore, the molecular mechanisms related to tumor formation and progression of T-LBL should be studied to provide effective therapies for its prevention.

The anaphase-promoting complex/cyclosome (APC/C) plays an important role in the process of protein degradation during mitosis (3). APC/C is significant in a variety of cellular events, including mitotic progression, controlling differentiation, etc. (4). Moreover, the aberrant expression of APC/C is relevant to cancer occurrence (5). Cell division cycle 27 (CDC27) is a critical subunit of APC/C that is in charge of binding to two coactivators: CDH1 and cell division cycle 20 (CDC20) (6). CDH1 and CDC20 have tumor suppressor function and oncogenic function, respectively (7–9). Although many studies have shown that CDC27 mutations are detected in various cancer specimens, the role of CDC27 in cancer is uncertain, especially in lymphoma (10).

Programmed death ligand-1 (PD-L1), as the ligand of programmed cell death protein 1 (PD-1), is a key immune checkpoint molecule that is frequently overexpressed in various tumors and correlates with tumor cells evading immune surveillance (11). Therefore, blocking the PD-1/PD-L1 axis is critical for cancer immunotherapy. However, the response rates to PD-1/PD-L1 immune checkpoint blockade therapy are likely to vary widely, ~10–20% across whole tumor types (12, 13). It is urgent to find predictive molecules and relevant pathways for PD-1/PD-L1 checkpoint blockade immunotherapy to develop innovative treatments.

In our previous studies, we found some prognostic clinical indicators in T-LBL (14, 15). We also found that the novel mutation in CDC27, which ranked highly in the samples (16). However, the function of relative targeted gene or therapy, such as CDC27, is not completely clear. In the present study, we aimed to determine whether CDC27 can be used as a target for T-LBL patients by analyzing 46 cases of T-LBL tissues and the T-LBL cells. The results offer new insights into the mechanisms of T-LBL tumorigenesis and suggest that the CDC27-PD-L1 axis could be used as a target in T-LBL treatment.

## Materials and Methods

### Cell Lines and Culture

Sup-T1 cells were purchased from American Type Culture Collection (Manassas, VA, USA). Jurkat and 293T cells were purchased From the Cell Bank of Type Culture Collection of the Chinese Academy of Sciences (Shanghai, China). 293T cell line was cultured in high glucose DMEM medium (Thermo Fisher Scientific), and the other cell lines were cultured in RPMI 1640 medium (Thermo Fisher Scientific). The cells were cultured in corresponding medium supplemented with 10% fetal bovine serum (Sigma-Aldrich, St. Louis, MO, USA), 100 U/mL penicillin, and 100 mg/mL streptomycin (Invitrogen, California, USA) in an incubator maintained at 37°C in a humidified atmosphere containing 5% CO_2_ and 95% air.

### Patient Tissue Samples and Clinicopathological Characteristics

Forty-six T-LBL tissues and 30 reactive hyperplasia of the lymph node tissues were collected in this study. All patients were histologically and clinically diagnosed between 2014 and 2019 in the First Affiliated Hospital of Zhengzhou University (Henan, China) and signed informed consent for the collection of tissues. The protocols were confirmed by the Institutional Research Ethics Committee of the First Affiliated Hospital of Zhengzhou University (Henan, China).

### Immunohistochemistry (IHC)

The detailed method has been described in a previous study (17). Sections were incubated with the primary antibodies overnight at 4°C, including CDC27 (Santa Cruz, CA, USA, sc-9972, 1:50), Ki67 (Abcam, MA, USA, ab15580, 1:100), and PD-L1 (Proteintech, Wuhan, China, 17952-1-AP, 1:100). The protein expression levels were evaluated semi-quantitatively according to the intensity and the percentage of staining area (18). The score criterion of intensity is as follows: <5%, – (negative = 0); 6–20%, + (weakly positive = 1); 21–50%, ++ (moderately positive = 2); >50%, +++ (strongly positive = 3). The scores were assessed by pathologists without prior knowledge of the patients' and the xenograft tumors' information. H-score that ranged from 0 (no staining) to 300 (maximum staining) were calculated with the following formula: 1 × (% light staining) + 2 × (% moderate staining) + 3 × (% strong staining).

### Stable Cell Line Construction

To overexpress CDC27, 12 μg purified pCDH- CMV-MCS- EF1- copGFP- T2A- puro- vector or pCDH- CMV- MCS- EF1- copGFP- T2A- puro- CDC27 plasmid was cotransfected with 6 μg pMDL-G/P-RRE, 6 μg pCMV-VSVG, and 7.5 μg pRSV-REV using Lipofectamine 6000 (Introgen, Carlsbad, CA, USA) according to the manufacturer's protocol. After 72 h, the viruses were harvested and used for transduction with 8 mg/ml polybrene. Stable CDC27 knockdown cell lines were generated using lentiviral constructs expressing short hairpin RNA (shRNA) of CDC27 (target sequence: shCDC27 #1 CAAGTACCTAATCATAGTTTA; shCDC27 #2 GAGCCAATAACCCAAGAAGAA) and negative control (target sequence: TTCTCCGAACGTGTCACGT), which were synthesized by Shanghai GeneChem (Shanghai, China). Cells were infected with lentivirus (multiplicity of infection = 50) by using polybrene (5 μg/ml) for 16 h.

### Cell Viability Assay

Cell proliferation was assayed by using the Cell Counting Kit-8 (CCK-8; Dojindo Laboratories, Tokyo, Japan) according to the manufacturer's protocol. First, T-LBL cells were seeded into 96-well-plates in triplicate (2 × 10^3^ cells per well) and totally cultured for 5 days. Then, the cells were incubated with a CCK-8 solution for 2 h at 37°C in an atmosphere containing 5% CO_2_. Absorbance was determined at OD 450 nm using a Multiskan FC microplate reader (Thermo Scientific, Waltham, MA, USA).

### Colony Formation Assays

In the colony formation assay (GENMED, SCIENTIFICS INC, USA), cells were seeded into 6-well-culture plates (1,000 cells per well) with Reagent A and incubated at 37°C for 14 days. Then, the cells were fixed and stained with 0.1% crystal violet solution (Amresco: C3886). The number of colonies containing more than 50 cells was counted.

### EdU-647 Labeling

1 × 10^6^ cells were seeded in 24-well-plates and labeled with 10 μM EdU (Click-iT™ Plus EdU Alexa Fluor™ 647 Flow Cytometry Assay Kit, lot: C10634) in 37°C for 2 h. After staining, fixation, and membrane permeability, cells were treated with 1 × Click-iT staining reaction solution for 30 min in darkness. After three washes, samples were added with 5 μl 7-AAD and analysis was performed on a flow cytometer (FACSCanto II, BD Bioscience, San Jose, CA, USA).

### Cell Cycle Analysis

Cells were harvested, washed, and fixed in precooled 75% ethanol mixed with phosphate buffer saline (PBS) at 4°C overnight. The next day, the cells were washed and resuspended in 500 μl RNase and propidium Iodide (PI) mixed solution from a cell cycle detection kit (KeyGen Biotech, KGA512), and the cells were incubated at 37°C for 30 min for staining. All cell samples were harvested and analyzed on a flow cytometer.

### Apoptosis Detection

Cell apoptosis was analyzed with an APC-annexin V Kit (BD Pharmingen). After 48 h of serum-starved culture, cells in each group were fully harvested and resuspended in annexin-V binding buffer. Next, cells were stained with APC-annexin V and 7-AAD Viability Staining Solution and incubated in the dark for 30 min at room temperature before being detected by a flow cytometer.

### Luciferase Gene Reporter Assay

The pGL3 plasmid containing the PD-L1 promoter region and luciferase reporter gene was purchased from GeneChem (Shanghai, China). The detailed procedures of the luciferase gene reporter assay were performed as previously described (19). According to the manufacturer's instructions, 24-well-plates were used to seed a total of 1 × 10^5^ 293T cells which were co-transfected with the corresponding plasmids, including the pGL3-luciferase plasmids (control vector or containing PD-L1 promoter), the indicated silencing transfection plasmids (shNC or shCDC27#1), and pRL-TK-Renilla vector. After 48 h, firefly and Renilla luciferase activities were measured and normalized by the Dual Luciferase Assay Kit (Promega, lot: 0000322867) according to the manufacturer's recommendations.

### Immunofluorescence Staining

The cells were fixed with 4% formaldehyde in PBS for 30 min at room temperature. After three washes with PBS, the cells were incubated in 0.25% Triton X-100 for 10 min to penetrate the membranes. To block the samples, the cells were treated with 5% bovine serum albumin in PBS for 1 h at room temperature. Then, the cells were incubated with the corresponding primary antibodies overnight at 4°C. Then the cells were washed and stained with a mixture of fluorescent secondary antibodies (Alexa Fluor 488 goat anti-mouse IgG, 1:100: Alexa Fluor 568 goat anti-rabbit IgG, 1:100, Life Technologies) and incubated for 1 h at room temperature in the dark. After three washes with PBS, the cells were stained with DAPI (sc-24941, Santa Cruz Biotechnology). Images were captured using a fluorescence microscope (DMI400B, Leica Company, Germany).

### Western Blotting Analysis

The detailed procedures of protein extraction and western blot analyses were previously described (20). Cells were washed and lysed in RIPA buffer with protease and phosphatase inhibitor cocktail (Thermo Scientific, Waltham, MA, USA). Protein samples were loaded and separated by 10% SDS-PAGE and then transferred to PVDF membranes. The membranes were then blocked with 5% non-fat milk in TBS/Tween (0.05% Tween-20 in TBS) for 2 h. Membranes were incubated with primary antibodies including CDC27 (Santa Cruz, sc-9972; 1:200), CDK4 (Proteintech, 11026-1-AP, 1:1,000), cyclin D1 (Proteintech, 26939-1-AP, 1:1,000), cleaved PARP (Cell Signaling Technology, #5625, 1:1,000), cleaved caspase 3 (Cell Signaling Technology, #9664, 1:1,000), PD-L1 (Abcam, ab205921, 1:1,000), and GAPDH (Proteintech, 60004-1-lg, 1:10,000) overnight at 4°C and then with secondary antibodies (anti-rabbit or anti-mouse; Proteintech) for 1 h at room temperature. To quantify the relative protein levels, the band images were gathered by using a ChemiDoc™ XRS+ system (Bio-Rad Laboratories, Hercules, CA, USA).

### Animal Experiment

Female BALB/c nude mice (4–5 weeks old, 15–18 g) were purchased from the GemPharmatech Company (Jiangsu, China). The nude mice were housed five per cage and fed a standard sterile laboratory diet under humidity and temperature-controlled conditions. Human tumor xenograft models were created by subcutaneous injection of 1.0 × 10^7^ Jurkat cells (sh-NC/sh-CDC27, *n* = 5) in 0.1 ml of PBS/Matrigel (1:1 mixture, BD Biosciences, CA, USA) into the flank regions of the mice; tumors were allowed to form for 5 weeks. During the experiments, the tumor burden, general conditions and weight of nude mice were monitored twice daily. After 35 days, the nude mice were euthanized. The tumor volumes and tumor weights were measured. Balb/c nude mice were handled and treated under animal laboratory conditions according to the approved guidelines of the Institutional Animal Care and Use Committee of the First Affiliated Hospital of Zhengzhou University (Henan, China).

### Statistical Analysis

Statistical analyses were performed using SPSS software version 19.0 (IBM Corp., Armonk, NY, USA) or GraphPad Prism version 7.01 (GraphPad Software, Inc., La Jolla, CA, USA). Data were expressed as the mean ± Standard Deviation (SD). Comparisons between two groups and among three groups were performed by using Student's *t*-test and one-way ANOVA, respectively. Pearson's correlation was used for correlation analysis. Protein expression and clinicopathological indicators were assessed using χ^2^-test. The Kaplan-Meier estimator method and log-rank test were used to analyze survival data. A value of *p* < 0.05 was considered statistically significant.

## Results

### CDC27 Is Overexpressed and Correlated With Progression in T-LBL

**Figure 1:**
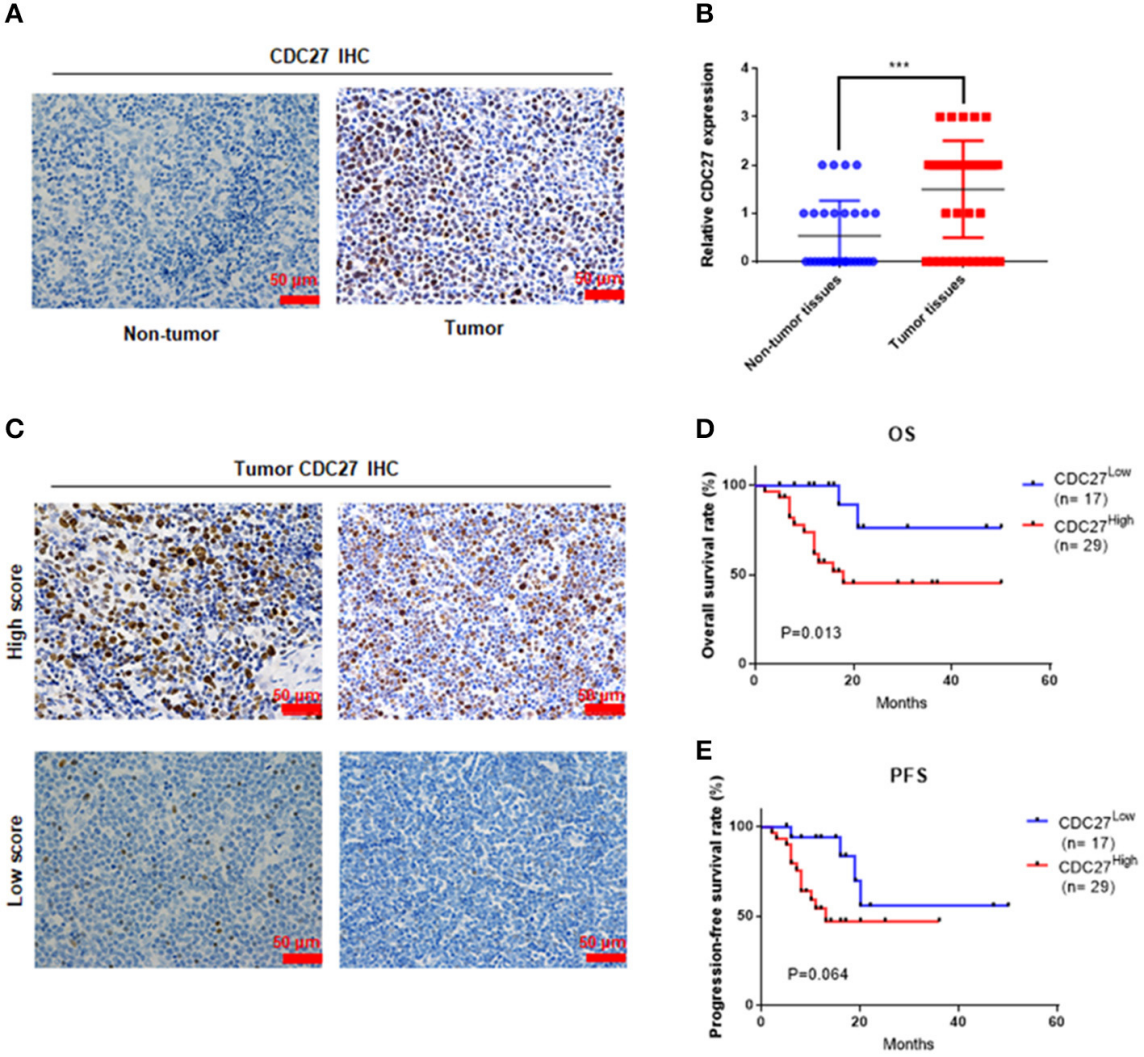
CDC27 is overexpressed in patient tumor samples and predicts decreased survival in T-LBL. **(A)** Representative images of CDC27 expression in T-LBL tissues (*n* = 46) and reactive hyperplasia (*n* = 30) of the lymph node tissues by IHC (×400 magnification). **(B)** Relative immunohistochemistry analysis for CDC27 expression T-LBL tissues and reactive hyperplasia of the lymph node tissues.-20 ***P < 0.001. **(C)** Representative images by IHC (×400 magnification) of T-LBL tissues which were divided into high score group (score = 2 or 3) or low score group (score = 0 or 1). **(D,E)** Kaplan-Meier analysis of OS and PFS in T-LBL patients.

Firstly, we used immunohistochemistry to evaluate CDC27 expression in tumor tissues from 46 T-LBL patients and 30 cases of reactive hyperplasia of the lymph node tissues. The results showed that CDC27 was mainly expressed in the nucleus. Compared with the reactive hyperplasia of the lymph node tissues, the tumor tissues had stronger staining intensity (Figures 1A,B). To analyze the relationship between CDC27 expression and the clinicopathological characteristics, we summarized the clinical information of the 46 cases of T-LBL in Table 1. There were 29 cases of tumor samples (63.1%) that were highly positive (Figure 1C). Further study showed that the expression of CDC27 had a significant correlation with the stage of disease (*p* = 0.014), which revealed that CDC27 expression may be associated with T-LBL progression. Then we investigated the correlation between CDC27 and the survival of T-LBL patients. The results of Kaplan-Meier survival analysis and log-rank tests in Figure 1D showed that T-LBL patients with high CDC27 expression exhibited significantly shorter overall survival (OS) than patients with low CDC27 expression (*p* < 0.01, hazard ratio = 5.182, CI = 1.871–14.35). And the correlation between CDC27 expression and progression free survival (PFS) was not statistically significant (*p* = 0.064, hazard ratio = 2.681, CI = 1.032–6.968; Figure 1E). The results demonstrated that high expression of CDC27 in T-LBL may be associated with poor prognosis to some extent.

**Table 1:** Clinicopathological findings and correlation with CDC27 expression in T-LBL.

**Variables**	**N (%)**	**CDC27-low (%)**	**CDC27-high (%)**	**χ^2^**	***P* value**
**Total cases**	46 (100%)	17 (36.9%)	29 (63.1%)		
**Gender**					
Male	34 (73.9%)	13 (28.3%)	21 (45.6%)	0.091	0.762
Female	12 (26.1%)	4 (8.7%)	8 (17.4%)		
**Age (years)**					
< 60	35(76.1%)	15 (32.6%)	20 (43.5%)	2.187	0.139
≥60	11(23.9%)	2 (4.3%)	9 (19.6%)		
**Stage**					
I-II	8 (17.4%)	6 (13.1%)	2 (4.3%)	6.016	0.014*
III-IV	38 (82.6%)	11 (23.9%)	27 (58.7%)		
**LDH increased**					
Y	13 (28.3%)	4 (8.7%)	9 (19.6%)	0.298	0.585
N	33 (71.7%)	13 (28.2%)	20 (43.5%)		
**Intrathoracic effusions**					
Y	31 (67.4%)	9 (19.6%)	22 (47.8%)	2.562	0.109
N	15 (32.6%)	8 (17.4%)	7 (15.2%)		
**Bone marrow**					
Y	28 (60.9%)	11 (23.9%)	17 (37.0%)	0.167	0.686
N	18 (39.1%)	6 (13.0%)	12 (26.1%)		
**ECOG**					
0–2	37 (80.4%)	16 (34.8%)	21 (45.6%)	3.208	0.073
3–5	9 (19.6%)	1 (2.2%)	8 (17.4%)		
**B symptoms**					
Y	17 (37%)	7 (15.2%)	10 (21.8%)	0.206	0.650
N	29 (63%)	10 (21.7%)	19 (41.3%)		
**Ki67**					
<80%	24 (52.2%)	10 (21.8%)	14 (30.4%)	0.478	0.489
≥80%	22 (47.8%)	7 (15.2%)	15 (32.6%)		
**IPI score**					
0-2	31 (67.4%)	11 (23.9%)	20 (43.5%)	0.088	0.766
3-5	15 (32.6%)	6 (13.0%)	9 (19.6%)		

*T-LBL T-cell lymphoblastic lymphoma; LDH, lactate dehydrogenase*;

* *P < 0.05*.

### CDC27 Promotes Cell Growth in T-LBL Cell Lines

**Figure 2:**
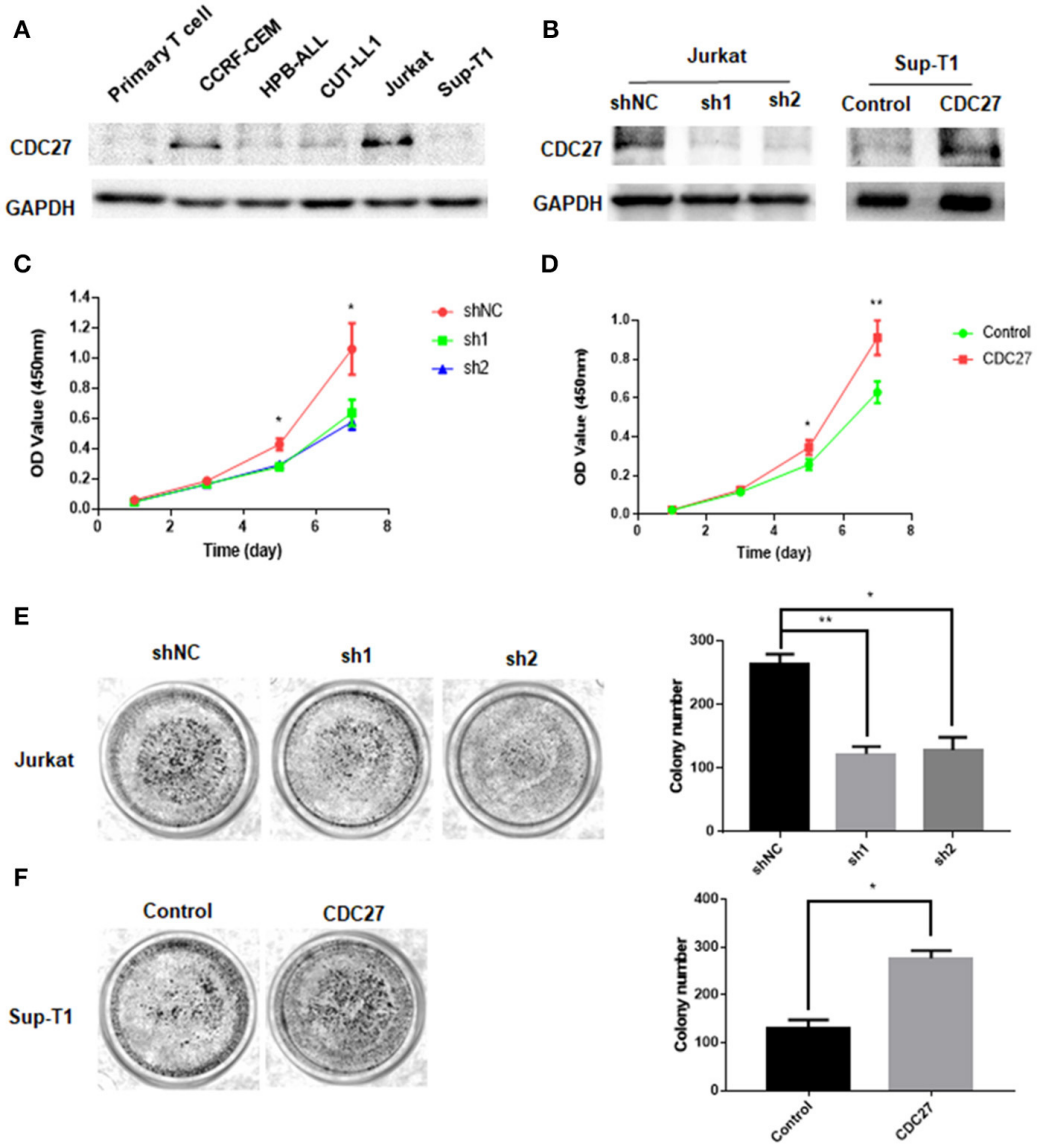
CDC27 promotes proliferation of CRC cells. **(A)** Protein expression was identified by western blotting analysis in T cell lymphoma cell lines. **(B)** The efficient overexpression or suppression of CDC27 was detected by western blotting. **(C,D)** Effects of CDC27 knockdown and over-expression on the proliferation of T-LBL cells (Jurkat and Sup-T1) *in vitro* by CCK-8 assay. *P < 0.05, **P < 0.01. **(E)** Colony formation number decreased under CDC27 knockdown in Jurkat cells. *P < 0.05, **P < 0.01. **(F)** CDC27 overexpression promoted cell growth in colony formation assays. *P < 0.05.

To test whether CDC27 alterations could affect the proliferation of T-LBL, we first examined the expression of CDC27 in five T cell lymphoma cell lines and primary normal T cells extracted from peripheral blood mononuclear cells using western blotting (Figure 2A). According to the results, we constructed stable CDC27-downregulated Jurkat cell lines that were transfected with shRNAs targeting CDC27 (sh1-CDC27, sh2-CDC27) or negative control shRNA (shNC-CDC27). In addition, we constructed a control Sup-T1 cell lines (control) and stable Sup-T1 cell line overexpressing CDC27 (CDC27). Western blotting was used to confirm the efficiency of knockdown and overexpression of CDC27 in these stable cell lines (Figure 2B). The results of the cell proliferation assay shown in Figures 2C,D indicated that the cell survival, or optic density (OD) value, was significantly decreased in the CDC27 shRNA-expressing Jurkat cells and increased in CDC27-overexpressing SUP-T1 cells. In addition, the sh-CDC27 group had weaker colony formation ability than the shNC group of Jurkat cells (Figure 2E). Furthermore, exogenous CDC27 expression also increased the colony formation capacity in Sup-T1 cells (Figure 2F). The results above indicated that CDC27 promotes cell growth in T-LBL.

### CDC27 Promotes G1/S Transition in the Cell Cycle

**Figure 3:**
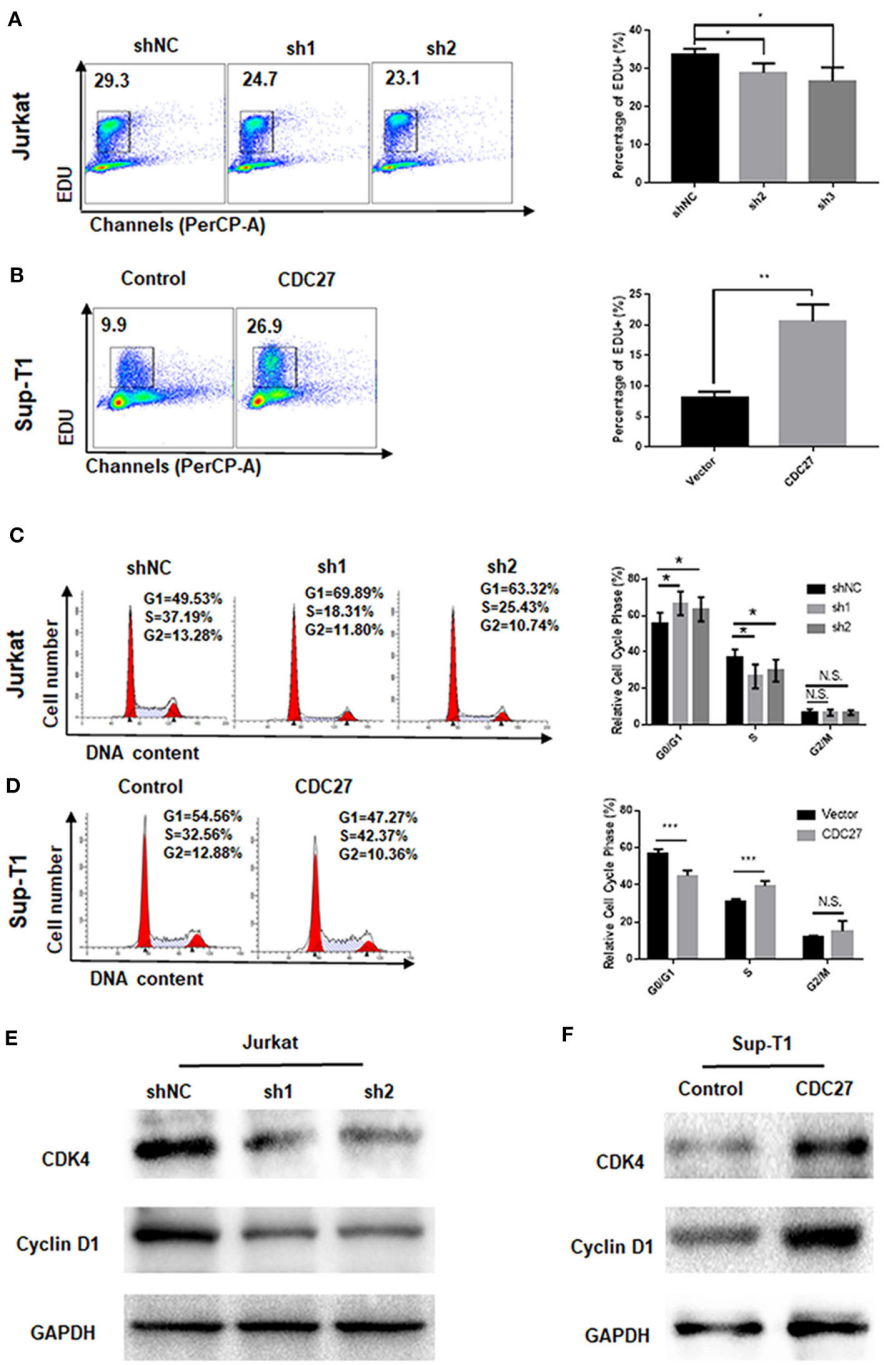
CDC27 influence the G1/S phase transition. **(A,B)** Cell proliferation were assessed by EdU incorporation assay. Data are representative of at least three independent experiments. **P < 0.01, *P < 0.05. **(C,D)** Flow cytometry was used to examine the cell cycle by PI staining of both Jurkat and Sup-T1 cells. Images and qualification of the cell cycle distribution in three independent experiments are shown. *P < 0.05, ***P < 0.001. N.S., not significant. **(E,F)** Western blot was performed to detect the expression levels of cell cycle related proteins in both Jurkat and Sup-T1 cells, respectively.

EdU cell staining and cell cycle detection assays were performed to verify whether the cell proliferation induced by CDC27 was related to cell cycle progression. The results of the EdU assay demonstrated that CDC27 knockdown decreased the S-phase proportion of Jurkat cells (Figure 3A). Overexpression of CDC27 promoted EdU synthesis in S-phase in Sup-T1 cells (Figure 3B). Furthermore, the results of the cell cycle assay showed that compared with Jurkat-shNC cells, Jurkat-shCDC27 had a significantly increased number of cells in G1 phase and a decreased the number of cells in S phase (Figure 3C). In stable Sup-T1 cells, CDC27 overexpression facilitated the G1/S transition (Figure 3D). Next, the level of G1/S-related cyclin D1 and CDK4 protein were detected by western blot. CDC27 knockdown decreased CyclinD1 and CDK4 expression (Figure 3E). In CDC27 overexpressing Sup-T1 cells, the expression level of CDK4 and cyclin D1 increased significantly (Figure 3F).

### CDC27-Knockdown Facilitates T-LBL Cell Apoptosis

**Figure 4:**
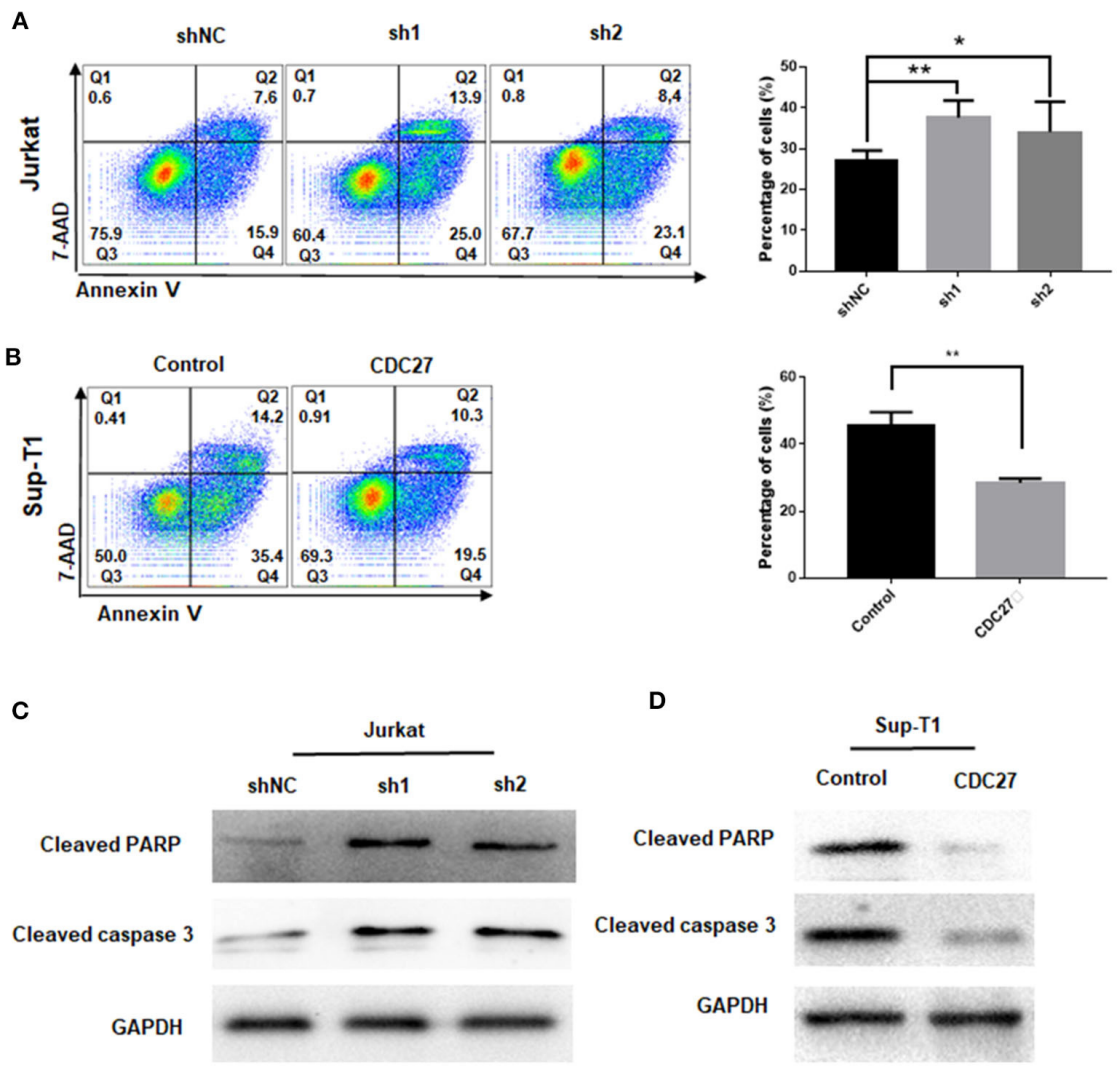
CDC27 inhibits cell apoptosis in T-LBL cells. **(A,B)** Flow cytometry was used to examine the apoptosis as the sum of both Q2 and Q4 quadrants (early + late apoptosis) by Annexin V/7-AAD staining of both Jurkat and Sup-T1 cells. Apoptosis rates were expressed as the mean (Q2 + Q4) ± SD of values from experiments performed in triplicate by using Student's *t*-test. *P < 0.05, **P < 0.01. **(C,D)** Western blot was performed to detect the expression levels of apoptotic related proteins in both Jurkat and Sup-T1 cells, respectively.

To clarify whether CDC27 knockdown influenced the apoptosis of T-LBL cell lines, Jurkat and Sup-T1 cells were starved for 48 h and subjected to Annexin V/7-AAD staining analysis. The percentage of apoptotic cells were counted as the sum of early apoptotic cells (quadrants Q4) and late apoptotic cells (quadrants Q2). The results showed that the proportion of apoptotic cells was significantly higher in CDC27 shRNA-expressing Jurkat and lower in CDC27 overexpressing SUP-T1 cells than in control cells (Figures 4A,B). We next verified the effect of CDC27 on several apoptosis-related proteins (Figures 4C,D). Given that PARP (poly ADP-ribose polymerase) is a kind of DNA repair enzyme that plays a significant role in DNA damage repair and apoptosis, the overexpression of CDC27 induced an obvious decrease in the levels of cleaved PARP. In addition, the expression of cleaved PARP remarkably increased in the shCDC27 group compared with the shNC group. The same trend in the expression of cleaved caspase 3 was also identified. These results suggested that CDC27 may influence the progression of T-LBL by regulating apoptosis.

### The Effect of CDC27 on PD-L1 Expression

**Figure 5:**
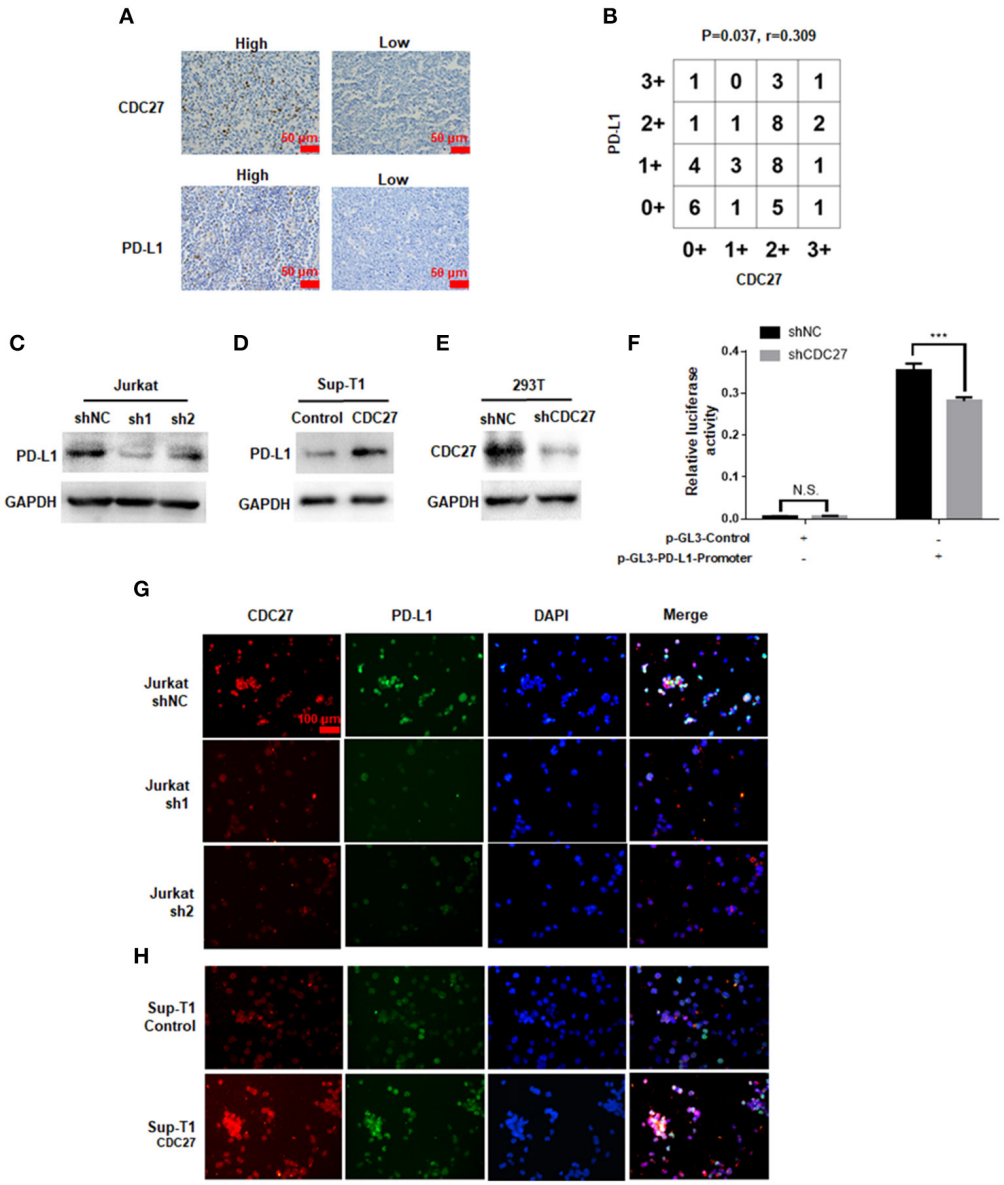
The effect of CDC27 on PD-L1 expression. **(A,B)** Representative IHC images and interaction plots of serial sections derived from patients with T-LBL (*n* = 46) were stained for CDC27 and PD-L1. **(C)** The protein expression of PD-L1 in shCDC27 Jurkat cells compared to control. **(D)** The protein expression of PD-L1 in CDC27 overexpression Sup-T1 cells compared to control. **(E)** The reduction of CDC27 protein levels in shCDC27-transfected 293T cells was detected by western blotting. **(F)** Luciferase reporter assays were performed to detect PD-L1 expression in the indicated cells with CDC27 suppressed. ***P < 0.001. N.S., not significant. **(G,H)** Immunofluorescence analysis of CDC27 and PD-L1 expression in Jurkat and Sup-T1 cells, respectively.

Given that APC/C^Cdh1^ can influence the expression of PD-L1 via degradation of SPOP, we wondered whether CDC27 can regulate PD-L1 in T-LBL cells (21). Firstly, we found a positive correlation between CDC27 and PD-L1 expression in human T-LBL tissues by IHC. The results showed that tissues with a higher score for CDC27 also had a higher level of PD-L1 expression (Figures 5A,B). There was an obvious positive correlation between CDC27 expression and PD-L1 expression in T-LBL tissues. In addition, the protein expression level of PD-L1 was significantly decreased in shCDC27 Jurkat cells compared with the control cells (Figure 5C). And in CDC27-overexpressing cells, the expression level of PD-L1 increased (Figure 5D). Moreover, 293T cells were transfected with luciferase reporter plasmids driven by the PD-L1 promoter (Figures 5E,F). Western blotting was used to confirm the efficiency of knockdown of CDC27 in 293T cells (Figure 5E). The results indicated that silencing CDC27 inhibited the activity of the PD-L1 promoter which demonstrated that CDC27 can regulate PD-L1 expression at transcriptional level at least in part (Figure 5F). We also found that PD-L1 protein expression was increased in high-CDC27 expression areas of the tumor cells by an immunofluorescence assay (Figures 5G,H). All the results suggested a positive correlation between CDC27 and PD-L1 in T-LBL.

### CDC27 Promotes Tumor growth in a Xenograft Mouse Model

**Figure 6:**
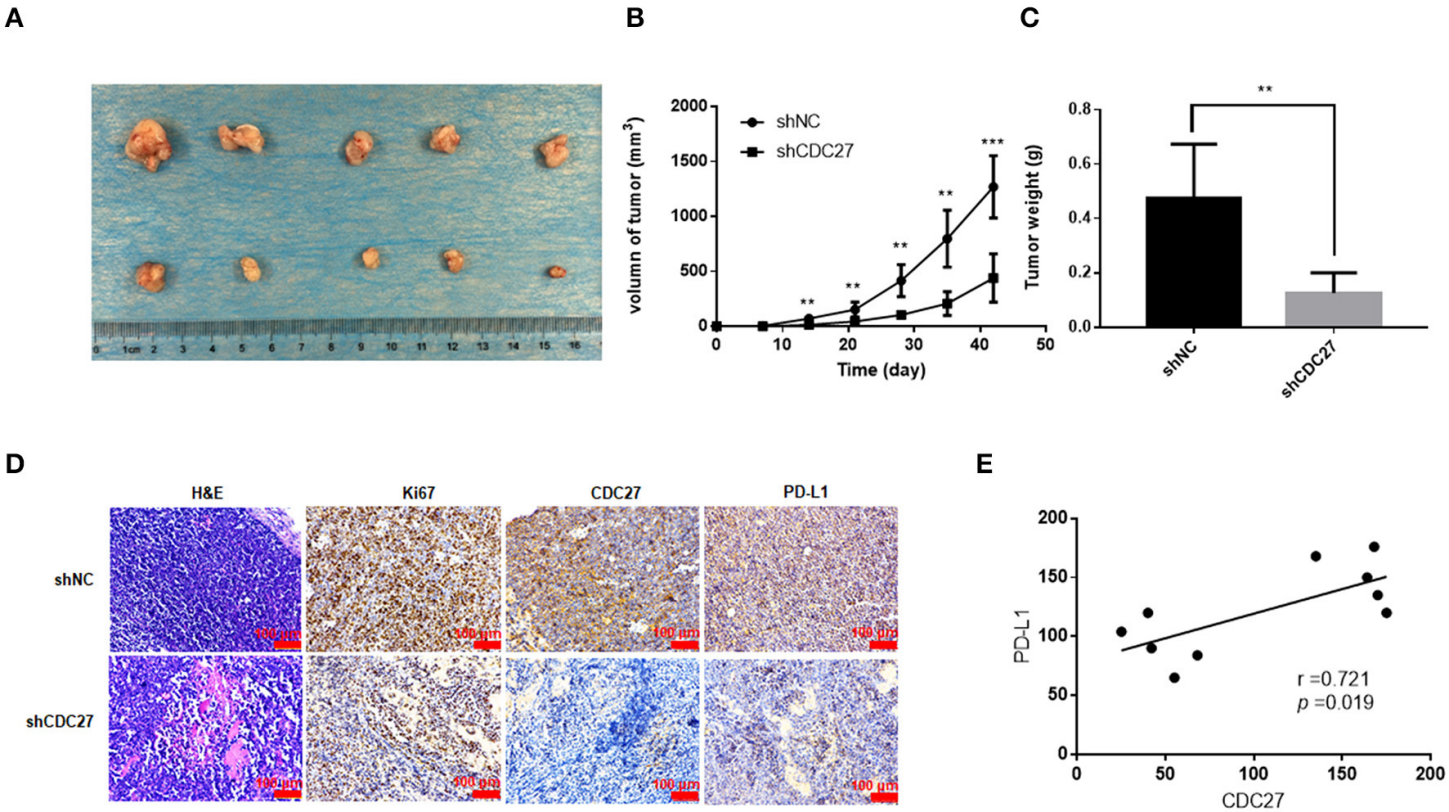
CDC27 promotes tumor growth *in vivo*. **(A)** Representative images of xenograft tumors Jukat cells with expression of shNC and shCDC27 (*n* = 5 in each group). **(B,C)** Tumor volume and tumor weight were analyzed. **P < 0.01, ***P < 0.001. **(D)** Representative H&E staining and immunohistochemistry staining of Ki67, CDC27, and PD-L1 in the xenograft tumor tissues (×200 magnification). **(E)** Correlations between CDC27 and PD-L1 expression in the xenograft tumor tissues were analyzed by Pearson's test.

Then, we used stable Jurkat cell lines to establish a BALB/c nude mouse xenograft model. ShNC or shCDC27 Jurkat cells were injected subcutaneously into female nude mice. Tumor volume was calculated according to the measured tumor size. After 5 weeks, the mice were killed and then the tumors were collected and weighed. The results showed that compared with the control group, knockdown of CDC27 in stable Jurkat cell contributed to a marked decrease in both tumor weight and volume (*n* = 5, Figures 6A–C). Next, we detected the expression of Ki67, which is a marker of nuclear cell proliferation, in excised mouse tumors by IHC. The staining intensity of Ki67 in the Jurkat-shCDC27 group was weaker than that in the control group (Figure 6D). In addition, the results of IHC also showed that the expression of PD-L1 had a positive correlation with CDC27 expression in tumors (Figures 6D,E). The results demonstrated that CDC27 is not only related to tumor proliferation but also affects PD-L1 expression in T-LBL.

## Discussion

The deregulation of APC/C has been reported to be connected with the genomic instability of tumor cells (22). As a core subunit of APC/C, CDC27 can bind CDH1 or CDC20, playing a key role in the process of recognizing and degrading target substrates. In recent years, several studies have reported that CDC27 can facilitate cell proliferation, migration and invasion in some types of cancers (23–25). Nevertheless, few studies indicated the opposite influence of CDC27 on cell functions and patients' survival analysis (26, 27). The expression and effect of CDC27 in lymphoma has not been reported or clarified. In our present study, CDC27 regulated the proliferation, apoptosis, and the cell cycle. By means of *in vitro* and *in vivo* experiments as well as clinical information analysis, we claimed that targeting CDC27 might offer new thoughts to the treatment of T-LBL.

Our study found that the expression of CDC27 was significantly unregulated in T-LBL tissues compared with the control tissues, which suggested that CDC27 may facilitate tumorigenesis of T-LBL and laid a foundation for further study. First, the proliferative effect of CDC27 was proven by cell viability assay and *in vivo* study. EdU-647 labeling assays and cell cycle assays revealed that the cancer-promoting function of CDC27 may be primarily because of the promotion of the G1/S phase transition. In addition, through the apoptosis detection test and western blotting experiments, we found that CDC27 could inhibit the apoptosis of T-LBL cells, which might be connected with the upregulation of related proteins in the apoptosis pathway. All of our functional studies in T-LBL cells demonstrated that CDC27 could promote tumor progression in various aspects. Intriguingly, Qiu et al. did not find evidence to prove that CDC27 is associated with apoptosis in colorectal cancer cells (23). This may be due to the tumor heterogeneity and more experiments are needed to confirm this point.

Although intensive chemotherapy has been applied in treating T-LBL patients, the 5-year OS is <67%, and the recurrence rate is over 30% (28). In this study, majority of T-LBL patients received regimens including dose-adjusted BFM-90 (prednisone, Vincristine, daunorubicin, L-Asaraginase, cyclophosphamide, cytarabine, bleomycin) or Hyper CVAD (cyclophosphamide, vincristine, doxorubicin, and dexamethasone, alternating with methotrexate and cytarabine). No research reported that the drugs mentioned above targeted CDC27. Our study revealed that CDC27 was overexpressed in T-LBL tumor tissues and correlated with OS. As for the negative results of PFS, it may be due to the limited number of cases that could be included in this study. Collectively, the detection of CDC27 expression could help to evaluate tumor staging and prognosis in T-LBL to a certain extent.

In recent years, immune checkpoint inhibition has been considered as a novel treatment method (29). PD-1 is one of the immune checkpoints responsible for regulating the immune response. Its ligand on tumor cells, PD-L1, can bind PD-1 and transmit suppression signals to T cells (30). Therefore, it is important to reverse immune escape by inhibiting the interaction between PD-1 and PD-L1. Nevertheless, most of patients fail to respond to PD-1/PD-L1 axis inhibitors. The abnormal expression of PD-L1 can be used as a predictive or prognostic marker of PD-1/PD-L1 inhibition therapy for patients (31–33). However, few studies have studied the expression and regulatory mechanisms of PD-1 or PD-L1 expression in T- LBL (34, 35). In this study, we first found a prominent positive relationship between tumor CDC27 and PD-L1 expression by IHC staining of T-LBL tissues. To further investigate and prove this connection, we also performed the western blotting, luciferase gene reporter assays and immunofluorescence staining, the results of which also proved our assumption that the expression of CDC27 is indirectly associated with PD-L1 in T-LBL tumor cells. However, the regulatory mechanisms between CDC27 and PD-L1 is uncertain, which may consist of a meshwork of proteins and pathways. A study of Zhang et al. showed that the activation of APC/C^Cdh1^ can promote the degradation of SPOP, which reduces ubiquitination-mediated PD-L1 degradation, resulting in increased PD-L1 levels (21). This study provided strong support that CDC27, as the most notable APC/C subunit, may participate in the regulation of PD-L1. Furthermore, Zhang et al. also indicated that CDK inhibitor plays a key facilitating role in the whole process (21). Recently, several studies indicated that CDK family had a close correlation with PD-L1 expression and immune escape (36–38). It gave clues that CDC27 may influence PD-L1 expression via modulating certain potential CDKs and their cell cycle or apoptosis related downstream molecules in the tumor microenvironment. Besides, previous studies have proved that TGF-β signaling can facilitate the activation of APC/C via CDC27, which allowing the transcription of genes necessary for growth inhibition and in turn leads to the transactivation of TGF-β-responsive genes for cell cycle arrest or apoptosis (39). Notably, the activation of TGF-β pathway can also induce the expression of PD-L1 and promote the occurrence of immunosuppression (33, 40, 41). Collectively, crosstalk among TGF-β signaling pathway may play a significant role in the regulation network of CDC27-PD-L1, which needs to be tested in the future.

The research findings above indicated that CDC27 may be involved in the progression and prognosis of T-LBL patients and is expected to become a promising novel target for the treatment. Based on current experimental basis, we hope that special inhibitors against CDC27 or its regulators can reactivate the anti-cancer effect of immune cells in T-LBL by reducing PD-L1 expression. And the CDC27 blockades can be designed and tested in conjunction with immune checkpoint inhibitors to overcome the therapeutic resistance. With the development of sequencing technologies, the personalized combination therapy will be feasible in the near future. A study showed that the pre-therapy tumor clone with mutations in CDC27 could not be detected after anti-PD-1 treatment in patients with hyperprogressive disease (42). In addition, CDC27 was also reported to be the most frequently mutated gene of resistant clones in a patient with exceptional response to immune checkpoint inhibitor (43). Therefore, it is worth to investigate whether the newly discovered “partner molecule” of PD-L1 can be served as a biomarker to predict the response of T-LBL patients to immunotherapy.

However, this study also has some limitations. Firstly, in recent years, CDC27 mutations have been found in some cancers (44–46). A study has identified mutated CDC27 as a tumor antigen which leaded to massive changes of chromosomal number (47). The mutated CDC27, which has not been investigated thoroughly by a conventional approach, may play an important role in cancer development. We tried to investigate the presence of alleles related to T-LBL. However, we have not found the key mutations of CDC27 that can influence the tumor progression or tumorigenesis until now. This need to be confirmed by rigorous experiments and larger cohort studies in the future. Secondly, because of the low incidence of T-LBL, the number of patients included in the study was limited. Some intriguing molecular genetics of T-LBL patients are not well-characterized, mainly due to the scarcity of samples. Hence, more researches with larger sample sizes are needed to verify our conclusions and explore novel fields. Thirdly, there may be various mediators and additional mechanisms involved in PD-L1 regulation by CDC27. Further studies are required to identify more target proteins in CDC27-PD-L1 axis.

## Conclusion

CDC27 is overexpressed in T-LBL tissues and influences the cell functions as well as PD-L1 expression, suggesting a novel insight into tumor progression and a new regulatory axis with potential therapeutic utility.

## Data Availability Statement

The datasets generated for this study are available on request to the corresponding author.

## Ethics Statement

The studies involving human participants were reviewed and approved by Institutional Research Ethics Committee of the First Affiliated Hospital of Zhengzhou University (Henan, China). Written informed consent to participate in this study was provided by the participants' legal guardian/next of kin. This animal study was reviewed and approved by Institutional Animal Care and Use Committee of the First Affiliated Hospital of Zhengzhou University (Henan, China). Written informed consent was obtained from the individual(s), and minor(s)' legal guardian/next of kin, for the publication of any potentially identifiable images or data included in this article.

## Author Contributions

MZ, YS, and ZL conceived the project. YS, WenS, and YW performed most of the experiments. QX and WeiS provided help for data analysis and curation. YS wrote the manuscript. ZL, JL, and MZ improved and revised this manuscript. All authors have read and agreed to the published version of the manuscript.

## Conflict of Interest

The authors declare that the research was conducted in the absence of any commercial or financial relationships that could be construed as a potential conflict of interest.

## Abbreviations

T-LBL: T-lymphoblastic lymphoma
CDC27: Cell division cycle 27
PD-L1: Programmed death ligand-1
NHL: non-Hodgkin lymphoma
ALL: acute lymphoblastic leukemia
APC/C: anaphase-promoting complex/cyclosome
CDC20: cell division cycle 20
PD-1: programmed cell death protein 1
PBS: phosphate buffer saline
IHC: Immunohistochemistry
OD: optic density
SD: standard deviation
OS: overall survival
PFS: progression free survival.
