# Corrigendum: CDC27 Promotes Tumor Progression and Affects PD-L1 Expression in T-Cell Lymphoblastic Lymphoma

**DOI:** 10.3389/fonc.2020.583698

**Published:** 2021-01-27

**Authors:** Yue Song, Wei Song, Zhaoming Li, Wenting Song, Yibo Wen, Jiwei Li, Qingxin Xia, Mingzhi Zhang

**Affiliations:** ^1^Department of Oncology, The First Affiliated Hospital of Zhengzhou University, Zhengzhou, China; ^2^The Academy of Medical Science of Zhengzhou University, Zhengzhou, China; ^3^Lymphoma Diagnosis and Treatment Center of Henan Province, Zhengzhou, China; ^4^Department of Pathology, The Affiliated Cancer Hospital of Zhengzhou University, Henan Cancer Hospital, Zhengzhou, China

**Keywords:** CDC27, T-cell lymphoblastic lymphoma, PD-L1, cell cycle, APC/C

In the original article, there was a mistake in **Figure 3** and **Figure 4** as published. In **Figure 3**, we put the wrong picture of EdU result in shNC group. In **Figure 4B**, we put the wrong picture of Apoptosis result in Control group. The correct **Figure 3** and **Figure 4** appear below. And in the Materials and Methods parts, we give a supplementary statement that cell lines used for Immunofluorescence were transfected with plasmid without GFP tag.

**Figure 3:**
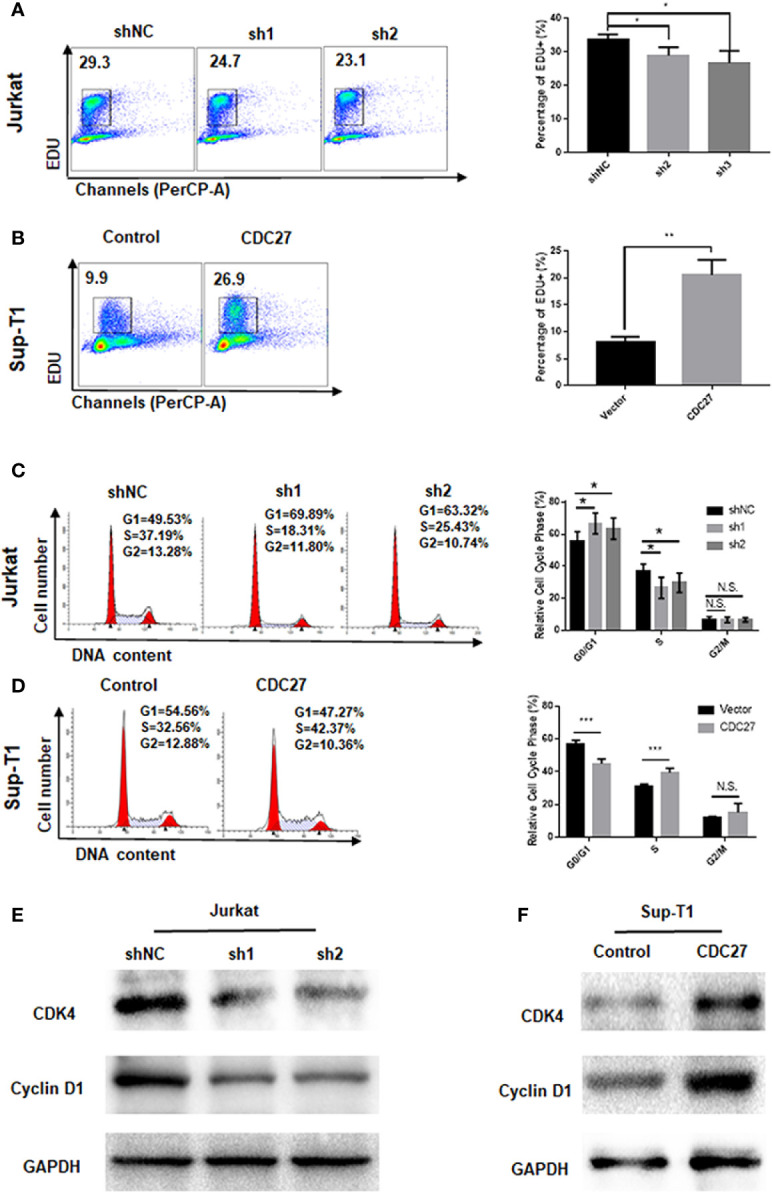
CDC27 influence the G1/S phase transition. **(A, B)** Cell proliferation were assessed by EdU incorporation assay. Data are representative of at least three independent experiments. **P < 0.01, *P < 0.05. **(C, D)** Flow cytometry was used to examine the cell cycle by PI staining of both Jurkat and Sup-T1 cells. Images and qualification of the cell cycle distribution in three independent experiments are shown. *P < 0.05, ***P < 0.001. N.S., not significant. **(E, F)** Western blot was performed to detect the expression levels of cell cycle related proteins in both Jurkat and Sup-T1 cells, respectively.

**Figure 4:**
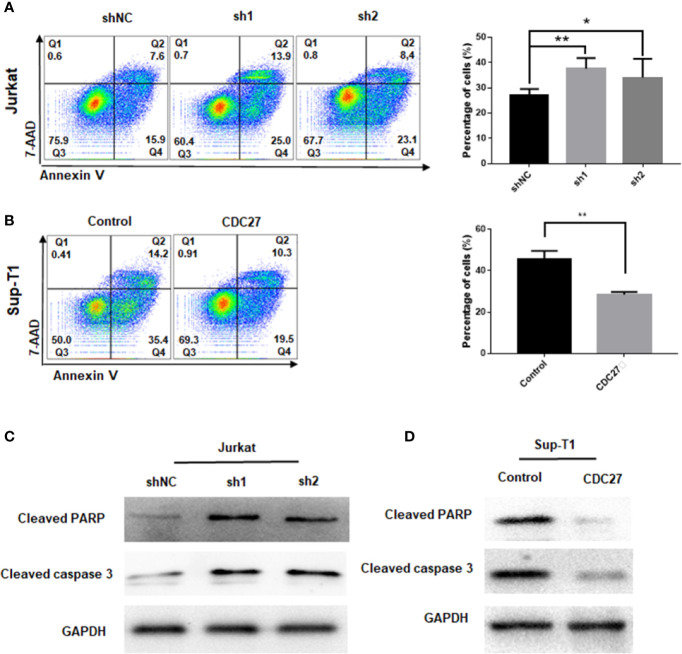
CDC27 inhibits cell apoptosis in T-LBL cells. **(A, B)** Flow cytometry was used to examine the apoptosis as the sum of both Q2 and Q4 quadrants (early + late apoptosis) by Annexin V/7-AAD staining of both Jurkat and Sup-T1 cells. Apoptosis rates were expressed as the mean (Q2 + Q4) ± SD of values from experiments performed in triplicate by using Student’s t-test. *P < 0.05, **P < 0.01. **(C, D)** Western blot was performed to detect the expression levels of apoptotic related proteins in both Jurkat and Sup-T1 cells, respectively.

The authors apologize for these errors and state that this does not change the scientific conclusions of the article in any way. The original article has been updated.

